# A Multiparametric Computational Algorithm for Comprehensive Assessment of Genetic Mutations in Mucopolysaccharidosis Type IIIA (Sanfilippo Syndrome)

**DOI:** 10.1371/journal.pone.0121511

**Published:** 2015-03-25

**Authors:** Krastyu G. Ugrinov, Stefan D. Freed, Clayton L. Thomas, Shaun W. Lee

**Affiliations:** 1 Department of Biological Sciences, University of Notre Dame, Notre Dame, Indiana, 46556, United States of America; 2 Center for Rare and Neglected Diseases, University of Notre Dame, Notre Dame, Indiana, 46556, United States of America; Tokyo Medical and Dental University, JAPAN

## Abstract

Mucopolysaccharidosis type IIIA (MPS-IIIA, Sanfilippo syndrome) is a Lysosomal Storage Disease caused by cellular deficiency of N-sulfoglucosamine sulfohydrolase (SGSH). Given the large heterogeneity of genetic mutations responsible for the disease, a comprehensive understanding of the mechanisms by which these mutations affect enzyme function is needed to guide effective therapies. We developed a multiparametric computational algorithm to assess how patient genetic mutations in SGSH affect overall enzyme biogenesis, stability, and function. 107 patient mutations for the SGSH gene were obtained from the Human Gene Mutation Database representing all of the clinical mutations documented for Sanfilippo syndrome. We assessed each mutation individually using ten distinct parameters to give a comprehensive predictive score of the stability and misfolding capacity of the SGSH enzyme resulting from each of these mutations. The predictive score generated by our multiparametric algorithm yielded a standardized quantitative assessment of the severity of a given SGSH genetic mutation toward overall enzyme activity. Application of our algorithm has identified SGSH mutations in which enzymatic malfunction of the gene product is specifically due to impairments in protein folding. These scores provide an assessment of the degree to which a particular mutation could be treated using approaches such as chaperone therapies. Our multiparametric protein biogenesis algorithm advances a key understanding in the overall biochemical mechanism underlying Sanfilippo syndrome. Importantly, the design of our multiparametric algorithm can be tailored to many other diseases of genetic heterogeneity for which protein misfolding phenotypes may constitute a major component of disease manifestation.

## Introduction

Sanfilippo syndrome is a lethal, hereditary neurodegenerative disease resulting from lysosomal accumulation of heparan sulfate and is one of the most prevalent classes of Lysosomal Storage Diseases (LSDs) [1–4]. Typically, LSDs are caused by a point mutation that disrupts the function of a single enzyme in the lysosome. As a result, unwanted metabolites accumulate in the lysosome, resulting in a broad range of symptoms [5]. Mucopolysaccharidosis type IIIA (MPS-IIIA) is a form of Sanfilippo syndrome resulting from a deficiency in functional N-sulfoglucosamine sulfohydrolase (SGSH, EC:3.10.1.1)—an enzyme involved in degradation of heparan sulfate [6,7]. Improper metabolic turnover of heparan sulfate in the lysosome leads to the severe neurological defects observed in MPS-IIIA patients. The first signs of the disease typically appear in the first to sixth year of life, and death occurs at a median age of 18 years [8].

At present, there is no effective treatment for MPS-IIIA disease. Current and emerging therapies include enzyme replacement therapy, substrate reduction therapy, gene therapy, and transplantation of gene-modified hematopoietic stem cells, with clinical trials established for all but substrate reduction therapy [9–14]. Very recent breakthroughs have shown some promise with targeted SGSH enzyme delivery across the blood brain barrier [15]. However, enzyme replacement therapy approaches have generally proven difficult, with immune system intolerance and enzyme delivery a significant concern. Additionally, enzymatic therapy strategies are costly, complicated, and involve high-risk procedures for patients, with therapeutic outputs that have only been shown to mitigate onset of new symptoms, underscoring the present need for novel approaches to treatment of LSDs [12,16].

Proper disease prognosis and clinical treatment is further complicated by the broad biochemical and clinical phenotype of the disease, which is a result of high genetic heterogeneity [8,17,18]. More than 100 missense mutations have been reported in the Human Gene Mutation Database (HGMD; www.hgmd.cf.ac.uk) for SGSH. Although some of these mutations have been shown to alter residues that 1. directly abrogate the active site of the enzyme or 2. result in the synthesis of a severely truncated enzyme, a large majority (87) of the documented SGSH mutations correspond to single amino acid changes that lead to enzyme impairments via an unknown mechanism.

To gain insight into the possible mechanisms by which a majority of MPS-IIIA mutations lead to changes in the activity of the SGSH enzyme, we conducted a comprehensive assessment of all documented MPS-IIIA mutations using a novel, multiparametric algorithm that evaluates the effect of a candidate mutation on overall protein quality and function. Specifically, our algorithm utilizes ten individual parameters to give a comprehensive predictive score of the protein stability and misfolding capacity of SGSH resulting from each of these mutations. The data presented herein demonstrate that a majority of the SGSH mutations that cause enzyme impairment are due to defects that impair proper folding of the three-dimensional conformation of the enzyme. Importantly, our algorithm gives a quantitative assessment of the severity of protein misfolding for a given patient mutation. This is especially pertinent within the context of pharmacological (chemical) chaperones, an emerging and highly promising therapy for the treatment of protein folding diseases. Pharmacological chaperones are small, bioactive molecules that can selectively bind to a target protein and stabilize the correct three-dimensional conformation throughout its biogenesis to result in a correctly folded functional protein. Indeed, chaperone-based approaches have been actively pursued for pathologies due to protein misfolding, such as Gaucher's disease, Nephrogenic diabetes insipidus, Alzheimer's Disease, Cystic Fibrosis, Parkinsons's Disease, and others [19–21].

A simple, yet crucial consideration in the development and therapeutic use of pharmacological chaperones is to assess which genetic mutations in a given disease population will be amenable to therapies to correct protein misfolding. Computational methods to analyze the effects of mutations that affect protein structure have been previously described [22–24]. However, many of these analyses assess only the contribution of a given mutation to the overall stability and three-dimensional structure of the protein. Currently, there are no predictive algorithms of protein biogenesis that incorporate a comprehensive analysis of protein instability with respect to specific and multiple parameters of cellular proteostasis. A multiparametric analysis would encompass a “beginning to end” look into all aspects of biogenesis to gain a more complete view of the impact of a given genetic mutation on protein maturation. These parameters would include important considerations of proteostasis such as translation rate, hydrophobicity and aggregation, posttranslational processing, and degree of evolutionary conservation. We propose that the multiparametric algorithm we have developed to evaluate genetic mutations in SGSH described in this report offers the most accurate and thorough predictive assessment of a given mutation to overall protein cellular dynamics, such that for a given patient mutation, pharmacological chaperone therapy can most appropriately be pursued.

Furthermore, the multiparametric algorithm that we describe not only provides the first comprehensive predictive assessment of a given genetic mutation to overall protein biogenesis, but also offers a generalized template such that any disorder for which a large heterogeneity of mutations contribute to a defect in protein function can be analyzed for chaperone therapy.

## Methods

### Scoring algorithm

To assess genetic mutations in SGSH using our multiparametric algorithm, we obtained the complete list of naturally occurring MPS-IIIA mutations from HGMD. Those genetic mutations that led to specific amino acid residue changes were selected for analyses. A total of 87 missense mutations in the MPS-IIIA gene were selected as appropriate candidates (Pre-selection criteria are described in Supporting Information).

The parameters for generating the comprehensive score of protein biogenesis were composed of individual assessments of biochemical, biophysical, and cellular features using *in silico* protein analytical programs. Structure related data were based on the crystal structure of N-sulfoglucosamine sulfohydrolase (PDB ID: 4MHX) [25]. Ten separate parameters were evaluated. Specifically, the amino acid residue change resulting from the corresponding genetic mutation was used to evaluate its effects on the following: 1. Translational rate; 2. Aggregation and hydrophobic propensity; 3. Stability; 4. Secondary structural motifs; 5. Proximity effects on the catalytic site; 6. Glycosylation; 7. Conformational flexibility and disulfide bonding; 8. Surface hydrophobicity and charge distribution; 9. Degree of conservation; 10. Physiological requirements for enzyme activity. Each parametric analysis was generally assigned a score for a given mutation of 0 or 1 (except the stability parameter where the maximum score was 2, see explanations below), with a score of 1 correlating with a negative effect of the mutation on the overall state of the protein. Therefore, total mutation scores can hypothetically range between 0 and 11. A general description of each parameter begins here; detailed methods for scoring each parameter are provided in the supplementary methods. Furthermore, the scoring analysis of one sample mutation is fully described in the supporting material (S1–S3 Figs.).

#### Parameter 1: Evaluation of protein translation rate

Early polypeptide conformations and folding trajectories are influenced by the *rate* of polypeptide synthesis [26–31]. The translation rate is affected by the distribution pattern of rare and common codons along the encoding mRNA sequence and by the abundance of tRNA species corresponding to these codons [32–37]. To assess how a given SGSH mutation can affect translation rate we compared the abundance of the tRNA species that correspond to the codons encoding the wild type and mutant residues.

#### Parameter 2: Evaluation of aggregation and hydrophobic propensity of the SGSH primary sequence

We evaluated and scored how a given amino acid mutation will affect the aggregative and hydrophobic propensities of the SGSH polypeptide. The *AGGRESCAN* algorithm was used to assess the effects of single amino acid changes on overall hydrophobicity and aggregation [38].

#### Parameter 3: Evaluation of the effect on SGSH protein stability

Proteins evolve to fold and perform their function in the crowded environment of the cell [39]. Each subcellular compartment of the eukaryotic cell comprises a specific set of macromolecules, small metabolites, and oxidizing conditions. Glycoproteins such as SGSH, which are co-post-translationally modified and targeted to specific organelles, are under constant dynamic stress owing to their changing subcellular environments [40]. Such proteins evolve to maintain delicate conformational equilibria through the dynamic process of folding and maturation. We evaluated and scored how a single residue mutation can affect SGSH stability, taking into account that destabilizing and stabilizing mutations can direct proteins to erroneous conformations [40,41]. The stability of a protein *in vivo* embraces the aspects of both thermodynamic stability and kinetic stability. Thermodynamic stability refers mainly to the difference in the energy states of a native (functional) and unfolded protein [41,42]. Kinetic stability refers to the size of the energy barrier which separates any two states of a cellular protein, for instance functional and non-functional [42–45]. Kinetic stability is of significant importance for the biogenesis of proteins which evolve to fold toward a functional state co-translationally [45–47]. Importantly, both thermodynamic and kinetic stability are affected by single point mutations and often represent the biophysical cause for protein malfunction [40,44,45,48]. This effect of disease-causing mutations is not surprising since both types of stabilities are intrinsically connected and a given mutation can cause a change in thermodynamic stability, which will lead to a change in kinetic stability, or affect both types in parallel [45]. Evaluating the precise mutational effect on kinetic stability *in vivo* of any given protein, including MPS-IIIA, is challenging because very few experimental methods for *in vivo* determination of the effect of the mutation on kinetic stability are available. Indeed, at this time, no computational methods exist that comprehensively address the effect of a disease-causing mutation on protein kinetic stability in the complex cellular environment [45,47,49,50]. In the current work, we evaluated the overall effect of point mutations on SGSH stability without distinction between thermodynamic and kinetic stability. We used a sequence-structure based computational algorithm (SVM), which was principally created and trained on a set of more than 3700 disease-causing point mutations from 243 proteins (http://www.snps3d.org) [48]. In addition, the method was evaluated and validated using sets of both disease and non-disease protein sequences. Hence, the SVM algorithm is a reliable tool for evaluating the effect of MPS-IIIA disease-causing mutations on protein stability for the purposes of our work. Since conformational stability is crucial in determining the folding pathway and biogenesis of a protein, this parameter was given higher weight than the other parameters (Table 1).

**Table 1:** Mutations scores.

Mutation/ Parameter	1. (0–1)	2. (0–1)	3. (0–2)	4. (0–1)	5. (0–1)	6. (0–1)	7. (0–1)	8. (0–1)	9. (0–1)	10. (0–1)	Total
**Ala30Pro**	0	0	2	1	1	0	1	0	1	1	**7**
**Gly33Arg**	0	0	1	0	1	0	1	1	0	0	**4**
**Tyr40Asn**	0	0	2	1	0	1	1	0	1	0	**6**
**Tyr40Ser**	1	0	2	1	0	1	1	0	1	0	**7**
**Asn42Lys**	1	0	2	0	0	1	1	1	1	0	**7**
**Ala44Thr**	0	0	0	0	0	1	0	0	0	0	**1**
**Leu59Phe**	1	0	1	1	0	0	1	0	0	0	**4**
**Ser66Trp**	0	1	2	0	0	0	1	0	0	0	**4**
**Ser68Arg**	0	0	2	0	1	0	1	1	1	0	**6**
**Thr79Pro**	0	0	2	0	0	0	1	1	1	0	**5**
**His84Tyr**	1	0	2	1	0	0	1	1	0	0	**6**
**His84Arg**	1	0	2	1	0	0	1	0	0	0	**5**
**Gln85Arg**	0	0	0	1	0	0	1	1	0	0	**3**
**Met88Thr**	0	0	1	0	0	0	0	0	1	0	**2**
**Gly90Arg**	0	0	2	0	0	0	1	1	0	0	**4**
**Ser106Arg**	0	0	2	0	0	0	1	1	1	0	**5**
**Thr118Pro**	0	0	2	1	0	0	1	1	1	0	**6**
**Gly122Arg**	0	0	2	1	1	0	1	1	1	0	**7**
**Pro128Leu**	1	1	2	0	1	0	1	0	0	0	**6**
**Val131Met**	0	0	0	0	0	0	0	0	0	0	**0**
**Thr139Met**	0	0	2	1	0	0	0	0	0	0	**3**
**Leu146Pro**	0	0	0	1	0	1	1	1	0	0	**4**
**Arg150Gln**	0	0	2	1	0	1	1	1	0	0	**6**
**Arg150Trp**	0	1	2	1	0	1	0	1	0	0	**6**
**Arg150Gly**	0	0	2	1	0	1	1	1	0	0	**6**
**Leu163Pro**	1	0	2	1	0	0	1	1	0	0	**6**
**Asp179Asn**	1	0	2	0	1	0	0	1	0	0	**5**
**Pro180Leu**	0	0	2	0	1	0	1	0	0	0	**4**
**Arg182Cys**	1	0	2	0	1	0	1	1	0	0	**6**
**Gly191Arg**	0	0	2	0	0	0	1	1	0	0	**4**
**Phe193Leu**	0	0	0	0	0	0	1	0	0	0	**1**
**Arg206Pro**	1	0	0	1	0	0	1	1	0	0	**4**
**Phe225Leu**	0	0	1	0	0	0	1	0	0	0	**2**
**Pro227Arg**	0	1	1	0	0	0	1	1	0	0	**4**
**Ala234Gly**	1	0	0	1	0	0	0	0	0	0	**2**
**Asp235Asn**	1	0	0	1	0	0	0	1	0	0	**3**
**Asp235Val**	1	1	2	1	0	0	0	1	0	0	**6**
**Arg245His**	1	0	2	1	0	0	1	0	0	0	**5**
**Arg245Met**	1	1	2	1	0	0	1	1	0	0	**7**
**Asp247His**	0	1	2	1	0	0	0	1	1	0	**6**
**Asp247Tyr**	0	1	2	1	0	0	1	1	1	0	**7**
**Gly251Ala**	0	1	2	1	0	0	0	0	0	0	**4**
**Thr271Met**	0	1	0	1	1	0	0	0	1	0	**4**
**Gly275Arg**	0	0	2	0	1	0	1	1	1	0	**6**
**Arg282Lys**	0	1	0	0	1	0	0	0	0	1	**3**
**Tyr286Ser**	1	0	2	0	0	0	1	0	0	0	**4**
**Pro288Ser**	0	0	0	1	0	0	1	0	0	0	**2**
**Pro288Leu**	1	1	2	1	0	0	1	0	0	0	**6**
**Glu292Lys**	0	0	2	1	0	0	1	1	0	0	**5**
**Pro293Ser**	0	1	2	1	0	0	1	0	1	0	**6**
**Pro293Thr**	0	1	2	1	0	0	1	0	1	0	**6**
**Ser298Pro**	0	0	1	0	0	0	1	1	0	0	**3**
**Glu300Val**	0	0	0	0	0	0	0	1	0	0	**1**
**Arg304Leu**	0	0	2	1	0	0	1	1	0	0	**5**
**Gln307Pro**	1	0	0	0	0	0	1	1	0	0	**3**
**Ala311Asp**	1	0	0	1	0	0	0	1	0	0	**3**
**Asp317His**	0	0	2	1	0	0	0	1	1	0	**5**
**Thr321Ala**	0	0	2	1	0	0	0	0	1	0	**4**
**Ile322Ser**	1	0	0	1	0	0	0	0	0	0	**2**
**Ser347Phe**	1	1	2	0	0	0	1	0	0	0	**5**
**Ser347Tyr**	1	1	2	0	0	0	1	0	0	0	**5**
**Ala354Pro**	0	0	0	0	0	0	1	1	0	0	**2**
**Glu355Lys**	0	0	0	0	0	0	1	1	1	0	**3**
**Ser364Arg**	0	0	2	1	0	0	1	1	0	0	**5**
**Glu369Lys**	0	0	2	0	1	0	1	1	0	0	**5**
**Tyr374His**	0	0	2	1	0	0	1	1	0	0	**5**
**Met376Arg**	1	0	2	1	0	0	1	1	0	0	**6**
**Arg377Cys**	1	0	2	1	0	0	1	1	0	0	**6**
**Arg377His**	1	0	2	1	0	0	1	0	0	0	**5**
**Arg377Leu**	0	0	2	1	0	0	1	1	0	0	**5**
**Gln380Arg**	0	0	0	1	0	0	1	1	0	0	**3**
**Leu386Arg**	0	0	2	1	0	0	1	1	0	0	**5**
**Asn389Lys**	1	0	2	1	0	0	1	1	0	0	**6**
**Asn389Ser**	1	1	2	1	0	0	0	0	0	0	**5**
**Leu411Arg**	0	0	0	1	0	0	1	1	0	0	**3**
**Thr415Pro**	0	0	0	1	0	1	1	0	0	0	**3**
**Thr421Arg**	1	0	0	0	0	0	1	1	0	0	**3**
**Arg433Gln**	0	0	2	0	0	0	1	1	1	0	**5**
**Arg433Trp**	0	1	2	0	0	0	0	1	1	0	**5**
**Asp440Gly**	0	0	2	1	0	0	0	1	0	0	**4**
**Asp444Gly**	0	0	2	0	0	0	1	1	0	0	**4**
**Glu447Lys**	0	0	2	0	0	0	1	1	0	0	**4**
**Arg456His**	1	0	0	1	0	0	1	0	0	0	**3**
**Gln472His**	0	0	1	1	0	0	0	1	0	0	**3**
**Asp477Glu**	1	0	2	0	0	0	0	1	0	0	**4**
**Val486Phe**	1	0	2	1	0	0	1	0	0	1	**6**

Mutations (first column) were scored according to ten parameters with potential score designations in parentheses. Column headings are as follows: 1. Translation Rate; 2. Aggregation Propensity; 3. Overall Cellular Stability; 4. Secondary Structure; 5. Catalytic Site; 6. Glycosylation; 7. Conformational Flexibility; 8. Surface Polarity/Change; 9. Evolutionary Conservation; 10. Physiological Function. The maximum possible score was 11. See Methods for detailed description of each scoring parameter.

#### Parameter 4: Evaluation of the effect on protein secondary structural motifs

As a human sulfatase, SGSH shares high sequence homology with the human arylsulfatases [51,52]. The arylsulfatases belong to the class of α/β proteins and are characterized by a three layer α/β/α fold [53]. Proper alignment of these structural elements is critical for correct formation of a functional catalytic site. Since each amino acid has a specific propensity to participate in secondary structure elements we evaluated and scored the involvement of mutated amino acid residues in these structural elements [54,55].

#### Parameter 5: Evaluation of residue mutation on proximity effects of the protein catalytic site

This evaluation was used to assess the relative contribution of the amino acid mutation on proximity effects that potentially perturb the catalytic active site of the protein.

#### Parameter 6: Evaluation of the glycosylation properties of the mutated residue

Glycosylation is a critical step in the proper maturation of known glycosylated proteins such as SGSH [56,57]. A given amino acid change can eliminate a known N-glycosylation recognition motif, or disrupt interactions with the glycosylating enzymes involved in posttranslational protein modification [58]. This parameter analyzed the potential alteration in glycosylation due to the amino acid change by a given mutation.

#### Parameter 7: Evaluation of the effect on conformational flexibility and disulfide-bond formation

Enzyme activity is inherently connected to protein dynamics and flexibility [59,60]. The precise location of key amino acids within discrete locations in the three-dimensional protein structure plays a critical role in protein flexibility. The unique conformational constraint of the proline side chain, and the ability of a proline residue to accommodate a *cis-/ trans*-conformation in proteins can contribute significantly to overall protein flexibility and function [61,62]. The structural features of a glycine residue and its lack of steric hindrance allow it to be a major contributor to increased protein flexibility [54,55]. Cysteine residues participate in disulfide bonding—an intramolecular feature critical for protein folding and stability [63,64]. In this analysis, any missense SGSH mutation involving changes in proline, glycine, or cysteine residues were noted for scoring.

#### Parameter 8: Evaluation of the effect on protein surface hydrophobicity and charge distribution

Substitution of a surface-exposed polar amino acid residue with a nonpolar residue increases the probability for erroneous protein interaction and aggregation [64]. Conversely, substituting a hydrophobic residue located within the core of the protein with a polar or charged residue is thermodynamically unfavorable [65]. Any charge distribution changes in the area of the catalytic site will affect the interactions with the negatively charged substrate of SGSH—heparan sulfate [66]. Finally, correct positioning of charged residues in the native structure of protein is important for correct formation of intramolecular salt bridges, which play an important role in protein stability [67]. The overall effect of the amino acid mutation on surface polarity and charge distribution was evaluated in this parameter.

#### Parameter 9: Evaluation of degree of evolutionary conservation of the selected amino acid change

Here we determined whether the amino acid mutation would occur in a position in the SGSH protein sequence that is evolutionarily conserved among its family of related proteins. Such conserved residues are likely to be important for function or stability. The evaluation was based on protein alignment of SGSH with 14 well characterized intracellular human sulfatases [52].

#### Parameter 10: Physiological requirements for enzyme activity

SGSH has been found to exist as a homodimer in crystal form, and a chelated calcium ion in the active site is thought to participate in catalytic mechanisms [25]. Thus, each mutation was evaluated for its role in proximity to the homodimer interface and Ca^2+^ coordination.

### Statistical Analysis

Analysis of the distribution of mutation scores was performed with GraphPad Prism software. Normality test was performed according D'Agostino-Pearson omnibus K2 algorithm, which accounts for the skewness (symmetry), and kurtosis (shape) of the Gaussian distribution [68]. The significance of the calculated skewness and kurtosis for the representative set was evaluated via calculation of Standard Error of Skewness (SES) and Standard Error of Kurtosis (SEK) [69]. The correlation analysis for the compound heterozygous scores was performed according the Spearman correlation test.

## Results

A total of 87 mutations were analyzed using our multiparametric algorithm for scoring the SGSH protein profile. All of the analyzed mutations are single amino acids changes in the SGSH protein coding region. For one mutation, Val226Ala, we were unable to find a consistent reference regarding the nature of the patient disease, and the mutation was therefore omitted from the analysis. The other 86 mutations represented 72 unique amino acid residue changes. Each mutation was analyzed individually as it represents a unique genotypic etiology of an individual MPS-IIIA patient. Each mutation was given a total evaluative score following an analysis of each of the ten individual protein parameters (Table 1). In our multiparametric algorithm, higher values for a given mutation correlated positively with the degree of impact this mutation would have on the overall proteostasis of the SGSH enzyme.

**Fig 1:**
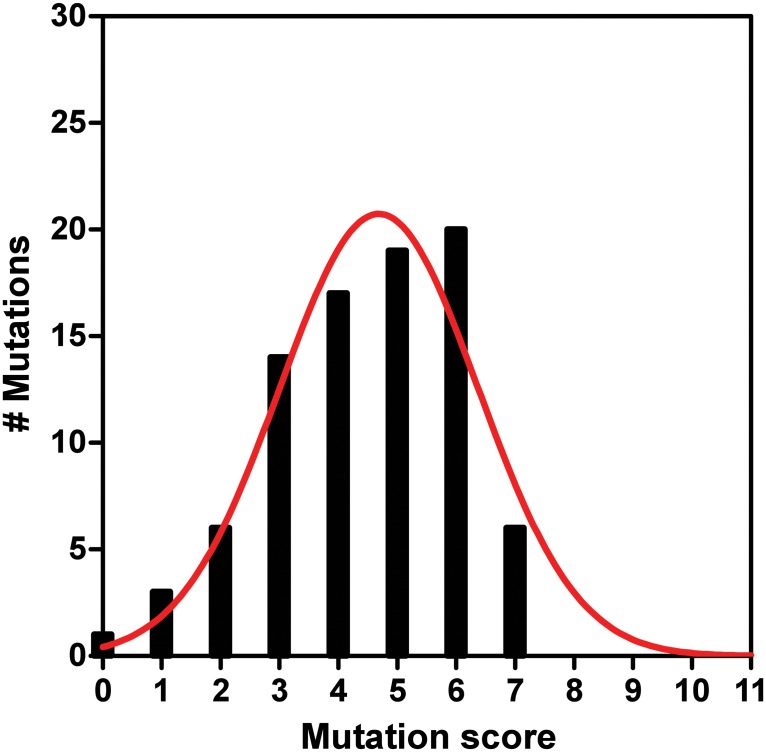
Total score distribution of all analyzed SGSH mutations described in the multiparametric evaluation study. The red curve represents the best data fit, indicating a Gaussian distribution. Distribution analysis, normality test and data fit were performed with GraphPad Prism software.

The SGSH mutations revealed a diverse score profile with total scores varying between 0 (one mutation) and 7 (Fig. 1 and Table 1). The total scores distribution passed the normality test. The normality test revealed a moderately skewed data set with a positive skew value of 0.683 (Table 2). The positive skew value, along with a lack of total mutation score greater than 7 suggests that mutations with high scores are highly unlikely, because mutations with such scores are lethal at an embryonic state and therefore not detected and described in the literature. To determine the likelihood that positive skewness is characteristic for the entire MPS-IIIA human population, but not a result of a biased data set, we weighed the skew value to the standard error of skewness (SES) (Table 2). The skew value was greater than two SES values (0.5194), which strongly suggests that the entire MPS-IIIA population is skewed positively according to our scoring [69]. An excess kurtosis value of negative 1.3770, which is greater than 2SEK (1.0278) (Standard Error of Kurtosis), indicates that the majority of the mutation scores are centered on intermediate scores and only few extreme (low or high) scores are present (Table 2). A Gaussian distribution fit to the scoring data demonstrates strong goodness of fit (R^2^ = 0.94, Fig. 1 and Table 3).

**Table 2:** Normality test and total mutational scores distribution.

D'Agostino & Pearson omnibus normality test	
K2	3.238
P value	0.1981
Passed normality test (α = 0.05)?	Yes
P value summary	ns
Skewness (S)	0.6830
SES (Standard Error of Skewness)	0.2597 (2SES = 0.5194)
**Skewness significance**	**Yes (S>2SES)**
Kurtosis (K)	-(1.377)
SEK (Standard Error of Kurtosis)	0.5139 (2SEK = 1.0278)
**Kurtosis significance**	**Yes (K>2SEK)**

Distribution analysis and normality were performed with GraphPad Prism software. Standard Error of Skewness (SES) and Standard Error of Kurtosis (SEK) were calculated according to [69].

**Table 3:** Gaussian fit of total mutation scores distribution.

Gaussian Best-fit values	
Amplitude	20.74
Mean	4.686
SD	1.682
Std. Error Amplitude	1.602
Mean	0.15
SD	0.1501
**95% Confidence Intervals**	
Amplitude	17.11 to 24.36
Mean	4.347 to 5.025
SD	1.342 to 2.021
**Goodness of Fit**	
Degrees of Freedom	9
R^2^	0.9355
Absolute Sum of Squares	45.9
Sy.x	2.258

Statistical analysis was performed with GraphPad Prism software.

The mutation scores distribution is characterized with mean value of 4.4 and standard deviation (SD) of 1.6. The clear divergence of the mean score value from 0 demonstrates that all analyzed mutations are expected to exhibit some effect on SGSH biogenesis and hence cause development of MPS-IIIA disease. Seventy of the mutations (~81%) have scores that fall within one SD of the mean value. These are mutations with scores between three and six. Such score values would be predicted to have a moderate effect on SGSH protein biogenesis. Six mutations have scores higher than one SD above the mean score value (score > 6), (Fig. 1 and Table 1). It can be predicted that these six mutations will exhibit much more pronounced effects on protein biogenesis and overall stability. The final ten mutations in our survey have scores that are lower than one SD below the mean score value (score < 3), (Fig. 1 and Table 1). These mutations are hypothesized to have milder effects on protein biogenesis and stability.

**Fig 2:**
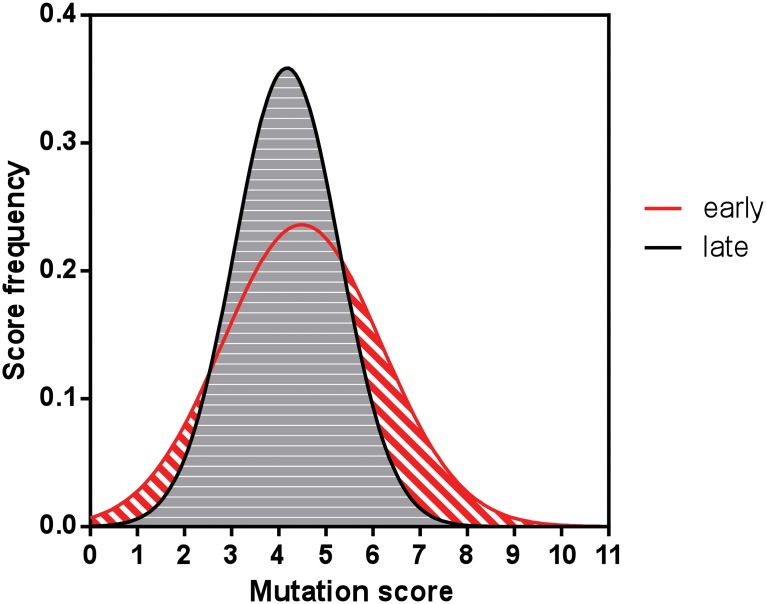
Distribution of total mutation scores according to age of disease onset. The data are presented as the fitted Gaussian curve and the area under the curve. Distribution analysis, normality test, and data fit were performed with GraphPad Prism software. Early and late ages of disease onset are according to [25] and the references therein. Early age of onset is considered less than 6 years of age. Late age of onset is considered greater than 6 years (see text for more information).

**Fig 3:**
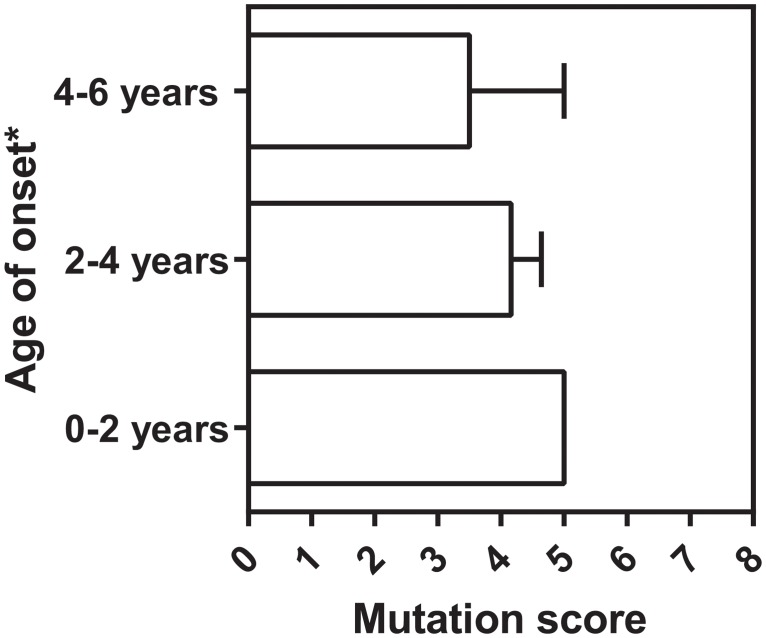
Relationship between total mutation score and MPS-IIIA age of onset in patients with homozygous genotype. Data is represented as column graph depicting mean value of scores for each group of patients. Error bars represent SEM (standard error). Each data point used for the calculation represents an individual patient (S1 Table). Only data for patients with clearly stated patient ID and severity phenotype are used. Age of onset must be interpreted carefully, because the literature cites age of disease diagnoses. This can be different from factual age of onset of the disease, as a correct diagnosis of rare diseases is often delayed.

Next we compared the distribution of the mutation scores in relation to the reported age of onset of MPS-IIIA patients [25]. Usually MPS-IIIA symptoms develop after birth. Clinical studies revealed that patients who develop a severe clinical phenotype have a disease age of onset varying between 1–6 years, whereas patients with mild clinical phenotype developed symptoms at ages older than 6 years with symptom development even in the second decade of life [2,8,70–73]. Our analysis shows that the mutation scores follow normal distribution for both patients with early and late age of disease onset (Fig. 2). The center of the mass of the scores was similar for both types of patients. However, the scores distribution for the patients with late age of onset was more skewed to low mutational scores. Notably, skew statistics based on SES calculations revealed that the skew value of the MPS-IIIA patient population with late age of onset was greater than 2SES values, suggesting that the trend to lower mutational scores for those patients is significant (Table 4). In contrast, the skew value of the MPS-IIIA patient population with early age of onset is less than 2SES values, suggesting that the trend to lower mutational scores is insignificant (Table 4). Further and more accurate analysis of the correlation between age of disease onset and mutational score requires comprehensive publication records where the exact genotype of a given patient is associated with clearly stated age of onset (or at least age of disease diagnosis). Unfortunately, due to the non-unified healthcare regulations in countries worldwide and the common difficulties of detecting and recording rare disease, such data are very limited. Our search through the literature revealed information for only eleven MPS-IIIA patients that bear a homozygous mutation and have clearly reported patient ID, age of onset, and SGSH genotype (S1 Table). Although all eleven patients have been recorded with an early age of disease onset we divided them into three age groups and analyzed the average mutation score for each group (Fig. 3). A general correlation between early age of onset and high mutational score was validated. Undoubtedly more data will be necessary for statistical justification of this trend, but our work proposes an organized model for future mutation documentation and analysis. Records for the age of disease onset of patients who are compound heterozygous for SGSH mutations were even more limited, and those data are not shown.

**Table 4:** Normality test of total mutation scores according to age of onset.

D'Agostino & Pearson omnibus normality test	Early age of onset (1–6 years)	Late age of onset (>6 years)
K2	2.128	5.659
P value	0.3451	0.059
Passed normality test (alpha = 0.05)?	Yes	Yes
P value summary	ns	ns
Skewness (S)	0.7681	1.424
SES (Standard Error of Skewness)	0.3978 (2SES = 0.7956)	0.4365 (2SES = 0.873)
**Skewness Significance**	**No (S<2SES)**	**Yes (S>2SES)**

Distribution analysis and normality were performed with GraphPad Prism software. Standard Error of Skewness (SES) was calculated according to [69].

In contrast to reports of age of onset, more extensive publication records report severity of MPS-IIIA symptoms and include a precise patient record and SGSH genotype. Classically, MPS-IIIA patients are divided into three clinical phenotypes—severe, intermediate, and mild (attenuated) [2,8,73]. Severe phenotypes are associated with severe central nervous system degeneration which causes general developmental delays encompassing speech delay, loss of cognitive functions and behavioral abnormalities. Such patients become completely dependent on supportive aid and usually die in the teenage years [8,72,73]. Patients with intermediate phenotypes have a slower rate of regression of intellectual and motor activities and live until young adulthood. Patients with mild phenotype develop disease symptoms at a significantly later age and maintain reasonable intellectual and motor activity. Their average age of death is well into adulthood [8].

**Fig 4:**
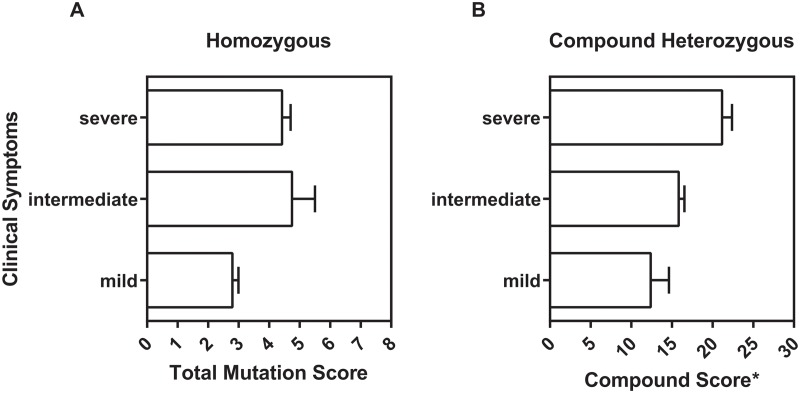
Relationship between total mutation score and severity of MPS-IIIA clinical symptoms. Data are represented as a column graph depicting scores mean value for (A) homozygous and (B) heterozygous patients. Error bars represent SEM (standard error). Each data point used for the calculation represents an individual patient (S2 and S3 Tables). Only data for patients with clearly stated patient ID and severity phenotype are used. See text for description of severe, intermediate and mild phenotypes. The total score for an individual compound heterozygous patient represents the product of the scores for each of the mutated alleles.

We have been able to identify twenty eight records for homozygous patients with clearly stated patient ID, SGSH genotype and classified clinical phenotype (S2 Table). The distribution of the mutation scores clearly correlates with the severity of the diseases—patients with low mutational scores tend to have milder clinical phenotype (Fig. 4A). Next, we analysed the correlation between mutation scores and disease severity for compound heterozygous patients (S3 Table). We explored two approaches to calculate the compound mutation score for such patients: (i) the compound mutational score was calculated as a sum of the scores of both mutations, and (ii) as a product of the scores of both mutations. In both cases the correlation between the compound score and the clinical phenotype was assessed with Spearman correlation. Both compound scores revealed significant (p<0.0001) positive correlations. However, the correlation with the compound score as a product of the two mutation scores yielded stronger correlations (S4 Table). Hence, the product of the score of the two mutations is the better predictor for MPS-IIIA disease severity for compound heterozygous patients (Fig. 4B).

## Discussion and Conclusion

Here we describe the first *in silico* multiparametric algorithm for the assessment of genetic mutations in SGSH proteostasis that utilizes a comprehensive panel of criteria involving all steps of protein biogenesis and maturation. Our direct interpretation of the method that we have developed is that it can be applied to an individual patient with a given genotype to predict disease severity outcome and evaluate the feasibility and suitability of a chaperone-based therapeutic approach for treatment.

We analyzed 86 mutations in the SGSH gene, which represent 2/3 of all patient-related MPS-IIIA disease-causing mutations annotated in HGMD. As such, our study represents the largest comprehensive meta-analysis of mucopolysaccharidosis type IIIA type mutations. Our current work specifically reveals for the first time that a large majority of SGSH mutations are likely to impede proper protein biogenesis, rather than to reduce activity of the completely folded, native protein (Fig. 1). These mutations therefore represent diseases due to protein misfolding rather than catalytic abatement, and thus are diseases with high probability of responding successfully to chaperone-based therapy [19,40,74–77]. *In vitro* studies already demonstrated that chaperone therapy could be effective to ameliorate the malfunction of mutated enzymes involved in mucoplysaccharidosis diseases such as MPS-IIIC [78]. Importantly, the list of the 86 mutations is inclusive of the most common mutations in MPS-IIIA patients: Ser66Trp, Arg245His and Ser298Pro. Hence, based on our analysis, chaperone-based therapies would likely be beneficial for the majority of the MPS-IIIA patients currently documented.

Our analysis clearly suggests that patient mutations with mild and late onset clinical phenotypes may correlate with mutations that have low scores in our algorithmic assessment (Figs. 3 and 4). Since a low score in our algorithm would indicate a mild defect in SGSH biogenesis, it is attractive to speculate that these patients with mild clinical phenotypes will be highly suitable for chaperone-based therapies. Moreover, some mutations with severe and early onset clinical phenotypes have an intermediate score, and may indeed be viable candidates for early intervention using chaperone therapies.

It is significant to note that our multiparametric algorithm provides considerable insight into the mechanisms through which each mutation affects MPS-IIIA biogenesis. Whereas some mutations affect common protein features as polypeptide stability and aggregation propensity, others affect SGSH-specific features such as the formation of unique structural elements characteristic for the class of protein sulfatases (Table 1). Such information may provide insights relevant to experimental planning and drug design.

We have demonstrated the utility of our algorithm using the genetic mutations described for Sanfilippo syndrome; however, we submit that the general principles underlying our algorithm can be modified to evaluate any disease involving protein misfolding for which a considerable heterogeneity in a given human mutation exists for the disease. We propose that the predictive score generated by our multidimensional protein biogenesis algorithm can therefore be integrated into an overall clinical evaluation program to select candidate genetic mutations that will best respond to pharmacological and chemical chaperone-based therapeutic approaches.

## Supporting Information

S1 Appendix Supplemental Methods and Scoring Example. A description of the MPS-IIIA patient mutation survey including detailed descriptions of all scoring criteria used for mutation analysis. A scoring sample analysis of the Arg245His mutation is described.
(DOCX)

S1 Fig SGSH protein structure model depicting Arg245His mutation. The β-strands are shown in yellow; α-helices are shown in red; turns/coils are shown in green; hydrogen bonding is shown as dotted green lines. (a) Residue Arg245 is presented as the space-filling model and boxed in white. (b) The native residue forms hydrogen bonds between its α-helix and the backbone of a nearby loop (c) which is absent in the R245H mutant. The model was obtained from the Research Collaboratory for Structural Bioinformatics (RCSB) Protein Data Bank (PDB) (PDB ID: 4MHX) [8,18]. The model incorporates residues 22–504 of the SGSH protein and was visualized with Swiss-PdbViewer 4.1.0.
(TIF)

S2 Fig Aggregation propensity profiles. (A) Aggregation propensity profile of full length SGSH. (B) Aggregation propensity profiles of wild type SGSH (gray), and Arg245His SGSH (red). For clarity only a region containing Arg245His mutation (red dot) is shown. (C) Aggregation propensity profiles of wild type SGSH (gray) and SGSH Ser66Trp (purple). For clarity only a region containing Ser66Trp mutation (purple dot) is shown. The square peaks in each profile represent the significant Hot Spot (HS) areas in the protein sequence. Profiles were created using the *AGGRESCAN* algorithm [6].
(TIF)

S3 Fig Sequence alignment of SGSH and related intracellular human sulfatases. Alignment was performed with ClustalX2 (default software color-coding was used). Only a small portion of the sequence alignment showing the relevant region for the amino acid residue Arg245 is shown for clarity. SGSH sequence is shown in the horizontal rectangle. The position of the Arginine at position 245 is indicated using a vertical rectangle. The annotation of the sulfatases is used as outlined in [14]. The stars denote residues of identity in all of the related protein sequences. Colons are used to indicate those amino acid positions where the residues show high conservation (amino acids with similar physico-chemical properties).
(TIF)

S1 Table Age of onset of patients homozygous for SGSH mutations. *Patient ID is according to the cited paper.
(DOCX)

S2 Table Disease severity of patients homozygous for SGSH mutations. If a mutation was referred to as mild/intermediate it was given an overall assessment of *intermediate*. If a mutation was referred to as intermediate/severe, it was given an overall assessment of *severe*.
*Patient ID is according to the cited paper.
** Severity is assumed from early death of patient caused by MPS-IIIA disease (12 years old) [27].
(DOCX)

S3 Table Severity of patients that are compound heterozygous for SGSH mutations. If a mutation was referred as mild/intermediate it was assigned a value of *intermediate*. If a mutation was referred as intermediate/severe, it was assigned a value of *severe*.
*Patient ID is according to the cited paper.**Severity is assumed from the current age at the clinical examination and the explanation of the reports for patients bearing S298P mutations (alive 36 years patient) [27].
^Data not included in analysis. Val131Met is the only mutation in our set that has a total score of 0, which does not allow calculations for compound heterozygous individuals.
(DOCX)

S4 Table Spearman correlation analysis of compound heterozygous patients. Analysis was performed with GraphPad Prizm software.
(DOCX)
