# Supplementary material for: A Multiparametric Computational Algorithm for Comprehensive Assessment of Genetic Mutations in Mucopolysaccharidosis Type IIIA (Sanfilippo Syndrome)
Source: PLoS One. 2015 Mar 25;10(3):e0121511. doi: 10.1371/journal.pone.0121511 (PMC4373678; doi:10.1371/journal.pone.0121511)
Supplement: S1 Appendix — A description of the MPS-IIIA patient mutation survey including detailed descriptions of all scoring criteria used for mutation analysis. A scoring sample analysis of the Arg245His mutation is described. (DOCX) [file pone.0121511.s001.docx]

**S1 Appendix**

**MPS-IIIA patient mutation survey**

All naturally occurring missense N-sulfoglucosamine sulfohydrolase (SGSH) mutations were obtained from The Human Gene Mutation Database (HGMD, http://www.hgmd.cf.ac.uk). As of Aug. 13, 2014, a total of 107 missense mutations have been annotated. Eleven mutations introduce a premature stop codon (nonsense mutations). Three mutations eliminate the initiation ATG codon. Two mutations were located in the signal sequence region of the protein. Four mutations altered residues in the conserved catalytic site of SGSH. These mutations either impede protein synthesis or result in synthesis of severely truncated polypeptide and therefore were excluded from our survey. The mutations located in the signal sequence region are likely to impede the initial targeting of the ribosome nascent chain complex to the translocon apparatus in the endoplasmic reticulum and also were excluded from further analysis [1-3]. As a result, a total of 87 SGSH missense mutations were selected for detailed analysis for the purposes of this study.

**Scoring criteria**

*Parameter 1: Evaluation of the effect on protein translation rate.* The tRNA abundance was expressed as the ratio of the tRNA gene copy number to the usage frequency of the corresponding codon. This ratio is defined as the normalized tRNA abundance. The SGSH gene codon sequences were obtained from HGMD database [4]. The tRNA gene copy numbers and the codon usage frequency for the human genome were obtained from the genomic tRNA database [5]. Scoring was performed according the following rules: (i) If only the codon encoding the wild type residue, but not the mutant residue is recognized by a tRNA through a wobble base-pairing (no tRNA gene copies for that codon), the effect of the mutation was scored with a value of <1>. (ii) If only the codon encoding the mutant residue, but not the wild type residue is recognized by a tRNA through a wobble base-pairing the mutation effect was scored with value of <1>. (iii) If both the codons of the wild type and the corresponding mutant residues are recognized by tRNA through a wobble-base pairing, the mutation effect was scored with value of <0>. (iv) If the normalized tRNA abundance value of the wild type residue is more than twice the difference (either lower, or higher) of the normalized tRNA abundance value of the mutant residue, the mutation effect was scored with value of <1>. (v) If the normalized tRNA abundance value of the wild type residue was less than twice the difference (either lower, or higher) of the normalized tRNA abundance value of the mutant residue, the mutation effect was scored with value of <0>.

*Parameter 2: Evaluation of the effect on aggregation and hydrophobic propensity of mutant SGSH primary sequences.* The *AGGRESCAN* algorithm was used [6]. The algorithm determines stretches of hydrophobic residues in the primary sequence of SGSH, which renders proteins prone to aggregation. The software creates a graphic output where any region with five or more consecutive amino acid residues with high aggregation propensity are designated as a Hot Spot (HS), (S2A Fig.) [6]. Aggregation propensity profiles were created for wild type SGSH sequences and all mutants. Next, the HS areas of the plots for each mutant sequence were compared to the HS areas of wild type SGSH sequence. Scoring of this parameter was performed according the following rules: (i) If a given mutation initiates the formation of new HS, the mutation was scored with value of <1>. (ii) If a given mutation residue increases the area of an already existent HS more than 20%, the mutation effect was scored with value of <1>. A threshold value of 20% was chosen in order to account only for mutations that significantly increase the aggregation propensity of the SGSH protein. Scores of zero were given in the following conditions: (i) If a given mutation residue increases the area of an already existent HS more with less than 20%; (ii) If a given mutation residue decreases the area of already existent HS, but does not eliminate it; (iii) If a given mutation does not create a HS that is missing in the wild type sequence, or (iv) If a given mutation eliminates a HS which previously existed in the wild type SGSH sequence.

*Parameter 3: Evaluation of the effect on SGSH protein stability.* Scoring was based on a support vector machine (SVM) approach to stability scoring of missense mutations hosted at http://www.snps3d.org [7]. For each amino acid substitution the algorithm calculates a score. A negative score value indicates that the mutation decreases protein stability. A positive score values indicates that the mutation increases overall protein stability. Scores above 0.5 or below -0.5 indicate greater statistical confidence. The scoring was performed according the following rules: (i) If a given mutation is predicted to decrease SGSH stability with a score lower than -0.5, the mutation effect was given a score with a value of <2>; (ii) If a given mutation is predicted to decrease SGSH stability with score between 0 and -0.5 (inclusive), the mutation effect was scored with value of <1>; (iii) If a given mutation is predicted to change SGSH stability with a prediction score above 0, the mutation effect was given a score value of <0>.

*Parameter 4: Evaluation of the effect on protein secondary structural motifs.* Scoring was based on the SGSH crystal structure model [8]. The scoring was performed according the following rules: (i) If a given mutation is located at an α-helix, 3_10_-helix, β-sheet, or extended β-sheet, the mutation effect was scored with value of <1>. (ii) If a given mutation is located at a turn or coil the mutation effect was scored with value of <0>.

*Parameter 5: Evaluation of residue mutation on* *proximity effects of the protein catalytic site.* Scoring was based on the primary amino acid sequence of SGSH and its structure model. The scoring was performed according the following rules: (i) If a given mutation is located within three amino acid residues from a catalytic site residue [9,10], the mutation effect was given a score value of <1>. (ii) If a given mutation side chain is located in within 6Å of a catalytic site residue (determined by Swiss-PdbViewer 4.1.0), and the mutation substitution involves the transition from a small side chain amino acid to one with a large side chain (amino acids: W, F, Y R, K), the mutation effect was scored with a value of <1> [11]. (iii) If none of the above rules applied, the mutation effect was scored with value of <0>.

*Parameter 6: Evaluation of the glycosylation properties of the mutated residue.* Scoring was based on the primary amino acid sequence of SGSH and its structure model. The scoring was performed according the following rules: (i) If a given mutation is located within three amino acid residues form a known site of glycosylation [12], the mutation effect was scored with value of <1>. (ii) If a given mutation is located within 6Å of a glycosylation site residue (determined by Swiss-PdbViewer 4.1.0), and the mutation substitution involves the transition from a small side chain amino acid to one with a large side chain (amino acids: W, F, Y R, K), the mutation effect was scored with value of <1>. (iii) If none of the above rules applied the mutation effect was given a score value of <0>. The specific mutation of amino acid Leucine to Proline at position 146 is the exception in this case as it is located between two adjacent glycosylation sites and is likely to have a significant impact on the glycosylation state of the SGSH protein. This specific mutation was therefore given a score value of <1> in this parameter.

*Parameter 7: Evaluation of the effect on conformational flexibility and disulfide-bond formation.* Scoring was based on the primary amino acid sequence of SGSH and its structure model. The scoring was performed according the following rules: (i) If a given mutation introduces a Cysteine residue, the mutation effect was scored with a value of <1>. (ii) If a given mutation introduces or eliminates a Proline residue, the mutation effect was scored with a value of <1>. (iii) If a given mutation introduces or eliminates the Glycine residue from a loop/turn region of the protein, the mutation effect was given a score value of <1>. (iv) If a given mutation results in the replacement of a given residue containing a small side chain with a residue containing a significantly large side chain (amino acids: W, F, Y, K, R) or vice versa, the mutation effect was scored with a value of <1>. (v) If none of the above rules applied, the mutation effect was given a score value of <0>.

*Parameter 8: Evaluation of the effect on protein surface hydrophobicity and charge distribution.* Scoring was based on the structure model of SGSH. The scoring of this parameter was performed according the following rules: (i) If a given mutation substitutes an uncharged amino acid residue with a charged residue or vice versa, the mutation effect was scored with a value of <1>. (ii) If a given mutation substitutes a negatively charged residue with a positively charged residue, or vice versa, the mutation effect was scored with a value of <1>. (iii) If a given mutation substitution was located in the buried portion of the protein (<30% solvent accessibility by Swiss-PdbViewer 4.1.0) and changed a hydrophobic or neutral residue with a hydrophylic residue, the mutation effect was scored with a value of <1> [13]. (iv) If a given mutation substitution involved the replacement of a surface-exposed (≥30% accessibility) hydrophilic or neutral residue with hydrophobic residue, the mutation effect was scored with value of <1>. (v) If none of the above rules applied, the mutation effect was given a score value of <0>.

*Parameter 9: Evaluation of degree of evolutionary conservation of the selected amino acid change.* This evaluation is based on the significance of a given mutated amino residue in relation to its overall degree of conservation in the specific protein family in which SGSH is classified. The scoring was based on the protein sequence alignment generated using the SGSH amino acid sequence and fourteen related, well-characterized intracellular human sulfatases [14]. The protein sequences were obtained from UniProtKB database and the alignment was performed with ClustalX2 (S3 Fig.) [15]. Based on this analysis, the scoring criteria of this parameter was as follows: (i) If a given mutated residue was highly conserved in more than one third of the sulfatases (six or more individual proteins), the mutation effect at that given position was deemed to be significant, and scored with a value of <1>. (ii) If a given mutated residue was conserved in more than one third of the sulfatases, but the mutation resulted in an amino acid substitution that was deemed to be of suitable similarity at the specific position, the mutation effect at that given position was scored with a value of <0>. (iii) If a given mutated residue was not conserved in more than one third of the sulfatases, the mutation effect at that given position was scored with a value of <0>.

*Parameter 10: Evaluation of effects of physiological requirements of enzyme activity.* Scoring was based on the structure model of SGSH, and reflected the requirement of N-sulfoglucosamine sulfohydrolase to chelate a calcium ion and its ability to form a homodimer [8]. (i) If the mutated residue was within three amino acid resudes of a residue involved in Ca^2+^ chelation, the mutation was given a score of <1>. (ii) If the mutated residue side chain was within 6Å of a side chain from the second subunit of the homodimer (determined by Swiss-PdbViewer), the mutation was given a score of <1>. (iii) If neither of the above rules applied, the mutation was given a score of <0>.

**Scoring example**

To illustrate how the scoring algorithm is applied here we described the analysis of the specific Arg245His mutation - one of the most common mutations in patients diagnosed with MPS-IIIA disease [16,17] (S1 Fig.). First, we assess if the mutation is likely to affect the SGSH translational rate (parameter 1, Table 1). In SGSH, the residue Arginine 245 is encoded by the mRNA codon CGC. In the human genome there is no tRNA species that recognize that specific codon through the classic Watson-Crick base-pairing. Rather, the CGC codon is recognized by a specific tRNA through wobble base-pairing [5]. Alternatively, the mutant Histidine residue is encoded by codon CAC, which is recognized by a cognate tRNA species with 11 gene copies in the human genome [5]. Therefore, according to our algorithm the Arg245His mutation will significantly influence the SGSH protein translation rate and the mutation was given a score value of <1> (Table 1). Next we evaluated if the mutation Arg245His will affect the aggregation and hydrophobic propensity of the SGSH polypeptide chain. We created aggregation propensity profiles for wild type and mutant SGSH sequences (S2 Fig.). The profile revealed that the mutation does not introduce any new hotspot (HS) protein areas, and only marginally increases the area of the existing adjacent HS (S2B Fig., red profile). This parameter therefore assigned the mutation Arg245His a score of <0>. For comparison, the specific residue Serine at position 66 is located in a distinct HS area. Introduction of the Ser66Trp mutation significantly increases the HS area surface (S2C Fig., purple profile). Therefore, the mutation Ser66Trp is expected to increase the aggregation propensities of the SGSH protein overall and the mutation is given a score of <1> based on this parameter (parameter 2, Table 1). According to www.snps3d.org the Arg245His mutation will decrease protein stability (confidence score of -1.02), so on the third parameter the mutation was scored with value of <2>. Arg245 is located in α-helix (S1 Fig.) in the three dimensional model structure of SGSH and therefore was scored <1> on the 4^th^ parameter. Arg245 is not in close proximity to any catalytic site residues or glycosylation site residues (S1 Fig.) and is not conserved among the human sulfatases (S3 Fig.). Therefore, the mutation was given a score of <0> on the 5^th^, 6^th^, and the 9^th^ parameters. According to our criteria Arg245His is expected to significantly affect overall protein conformational flexibility and scored <1> on the 7^th^ parameter. Arg245His is not expected to significantly alter charge and hydrophobicity and scored <0> on the 8^th^ parameter, and it has no effect on calcium chelation or homodimerization, resulting in a score of <0> on the 10^th^ parameter. Taken together, the multiparametric analysis of the SGSH mutation Arg245His assigned this mutation a total cumulative score of 5 out of 11 (Table 1).

**Supplemental References.**

1. Johnson, A.E., and van Waes, M.A. The translocon: a dynamic gateway at the ER membrane. Annu Rev. Cell. Dev. Biol. 1999;15: 799-842.
2. Rapoport, T.A. Protein translocation across the eukaryotic endoplasmic reticulum and bacterial plasma membranes. Nature. 2007;450: 663-669.
3. Boyd, R.E., Lee, G., Rybczynski, P., et al. Pharmacological Chaperones as Therapeutics for Lysosomal Storage Diseases. J Med Chem. 2013;56(7): 2705-25.
4. Cooper, D.N., and Krawczak, M. Human Gene Mutation Database. Hum Genet. 1996;98: 629.
5. Lowe, T.M., and Eddy, S.R. tRNAscan-SE: a program for improved detection of transfer RNA genes in genomic sequence. Nucleic Acids Res. 1997;25: 955-964.
6. Conchillo-Sole, O., de Groot, N.S., Aviles, F.X., et al. AGGRESCAN: a server for the prediction and evaluation of "hot spots" of aggregation in polypeptides. BMC Bioinformatics. 2007;8: 65.
7. Yue, P., Li Z., and Moult J. Loss of Protein Structure Stability as a Major Causative Factor in Monogenic Disease. J Mol Biol. 2005;353: 459-473.
8. Sidhu NS, Schreiber K, Pröpper K, Becker S, Usón I, Sheldrick GM, et al. Structure of sulfamidase provides insight into the molecular pathology of mucopolysaccharidosis IIIA. Acta Crystallogr D Biol Crystallogr. 2014;70(Pt 5): 1321-35.
9. Waldow, A., Schmidt, B., Dierks, T., et al. Amino acid residues forming the active site of arylsulfatase A. Role in catalytic activity and substrate binding. Journal of Biological Chemistry. 1999;274: 12284-12288.
10. Obaya, A.J. Molecular cloning and initial characterization of three novel human sulfatases. Gene. 2006;372: 110-117.
11. Guex N, Peitsch MC. SWISS-MODEL and the Swiss-PdbViewer: an environment for comparative protein modeling. Electrophoresis. 1997;18(15): 2714-23.
12. Di Natale, P., Vanacore, B., Daniele, A., et al. Heparan N-sulfatase: in vitro mutagenesis of potential N-glycosylation sites. Biochem Biophys Res Commun. 2001;280: 1251-1257.
13. Monera, O.D., Sereda T.J., Zhou N.E., et al. Relationship of sidechain hydrophobicity and alpha-helical propensity on the stability of the single-stranded amphipathic alpha-helix. J Pept Sci. 1995;1: 319-239.
14. Diez-Roux, G., and Ballabio, A. Sulfatases and human disease. Annual Review of Genomics and Human Genetics. 2005;6: 355-379.
15. Larkin, M.A. Blackshields, G. Brown, N.P., et al. Clustal W and Clustal X version 2.0. Bioinformatics. 2007;23: 2947-2948.
16. Valstar, M.J., Ruijter, G.J., van Diggelen, O.P., et al. Sanfilippo syndrome: a mini-review. Journal of Inherited Metabolic Disease. 2008;31: 240-252.
17. Yogalingam, G., and Hopwood, J.J. Molecular genetics of mucopolysaccharidosis type IIIA and IIIB: Diagnostic, clinical, and biological implications. Human Mutation. 2001;18: 264-281.
18. Arnold, K., Kiefer, F., Kopp, J., et al. The Protein Model Portal. J Struct Funct Genomics. 2009;10: 1-8.
19. Perkins KJ, Muller V, Weber B, Hopwood JJ. Prediction of Sanfilippo phenotype severity from immunoquantification of heparan-N-sulfamidase in cultured fibroblasts from mucopolysaccharidosis type IIIA patients. Mol Genet Metab. 2001;73(4): 306-12.
20. Weber B, Guo XH, Wraith JE, Cooper A, Kleijer WJ, Bunge S, Hopwood JJ. Novel mutations in Sanfilippo A syndrome: implications for enzyme function. Hum Mol Genet. 1997;6(9): 1573-9.
21. Emre S, Terzioğlu M, Coşkun T, Tokath A, Ozalp I, Müller V, Hopwood J. Biochemical and molecular analysis of mucopolysaccharidoses in Turkey. Turk J Pediatr. 2002;44(1): 13-7.
22. Di Natale, P., Balzano, N., Esposito, S., et al. Identification of molecular defects in Italian Sanfilippo A patients including 13 novel mutations. Hum Mutat. 1998;11: 313-320.
23. Beesley, C.E., Young, E.P., Vellodi, A., et al. Mutational analysis of Sanfilippo syndrome type A (MPS IIIA): identification of 13 novel mutations. J Med Genet. 2000;37: 704-707.
24. Montfort, M., Vilageliu, L., Garcia-Giralt, N., et al. Mutation 1091delC is highly prevalent in Spanish Sanfilippo syndrome type A patients. Human Mutation. 1998;12: 274-279.
25. Esposito S, Balzano N, Daniele A, Villani GR, Perkins K, Weber B, Hopwood JJ, Di Natale P. Heparan N-sulfatase gene: two novel mutations and transient expression of 15 defects. Biochim Biophys Acta. 2000;1501(1): 1-11.
26. Blanch L, Weber B, Guo XH, Scott HS, Hopwood JJ. Molecular defects in Sanfilippo syndrome type A. Hum Mol Genet. 1997;6(5): 787-91.
27. Valstar, M.J. Neijs, S. Bruggenwirth, H.T., et al. Mucopolysaccharidosis type IIIA: clinical spectrum and genotype-phenotype correlations. Ann. Neurol. 2010;68: 876-887.
28. Chabas, A., Montfort, M., Martinez-Campos, M., et al. Mutation and haplotype analyses in 26 Spanish Sanfilippo syndrome type A patients: possible single origin for 1091delC mutation. Am J Med Genet. 2001;100: 223-228.
29. Muschol N, Storch S, Ballhausen D, Beesley C, Westermann JC, Gal A, et al. Transport, enzymatic activity, and stability of mutant sulfamidase (SGSH) identified in patients with mucopolysaccharidosis type III A. Hum Mutat. 2004;23(6): 559-66.
30. Bunge S, Ince H, Steglich C, Kleijer WJ, Beck M, Zaremba J, et al. Identification of 16 sulfamidase gene mutations including the common R74C in patients with mucopolysaccharidosis type IIIA (Sanfilippo A). Hum Mutat. 1997;10(6): 479-85.
