# Supplementary figures and images for: A Multiparametric Computational Algorithm for Comprehensive Assessment of Genetic Mutations in Mucopolysaccharidosis Type IIIA (Sanfilippo Syndrome)
Source: PLoS One. 2015 Mar 25;10(3):e0121511. doi: 10.1371/journal.pone.0121511 (PMC4373678; doi:10.1371/journal.pone.0121511)

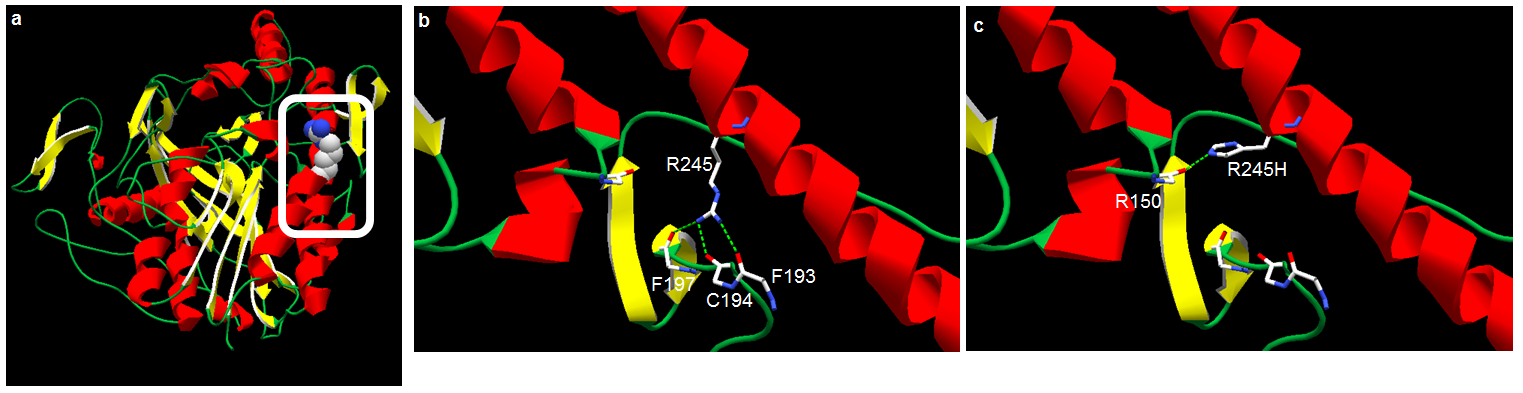

Supplement: S1 Fig — The β-strands are shown in yellow; α-helices are shown in red; turns/coils are shown in green; hydrogen bonding is shown as dotted green lines. (a) Residue Arg245 is presented as the space-filling model and boxed in white. (b) The native residue forms hydrogen bonds between its α-helix and the backbone of a nearby loop (c) which is absent in the R245H mutant. The model was obtained from the Research Collaboratory for Structural Bioinformatics (RCSB) Protein Data Bank (PDB) (PDB ID: 4MHX) [8,18]. The model incorporates residues 22–504 of the SGSH protein and was visualized with Swiss-PdbViewer 4.1.0. (TIF) [file pone.0121511.s002.tif]

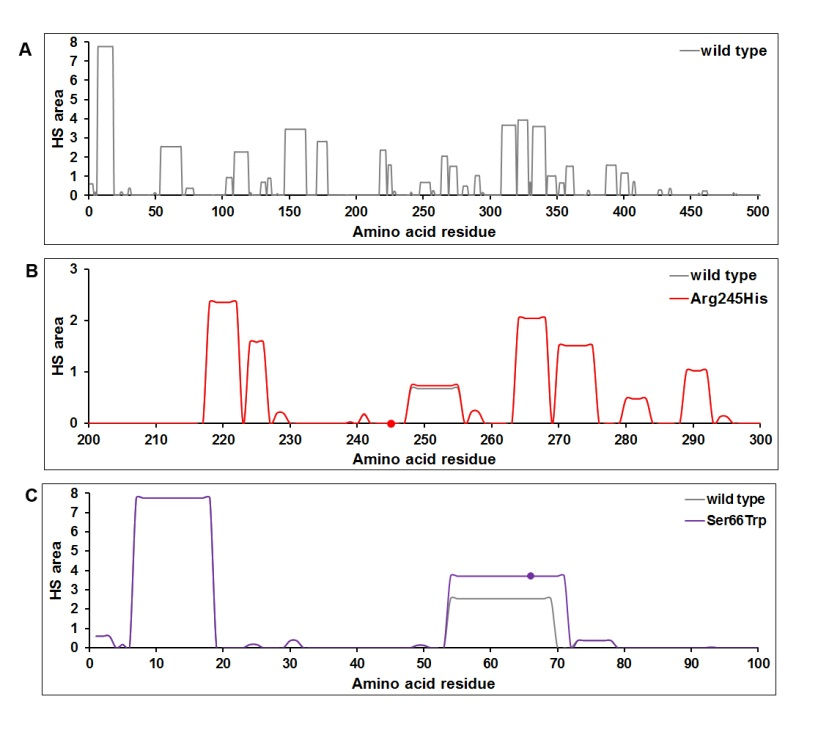

Supplement: S2 Fig — (A) Aggregation propensity profile of full length SGSH. (B) Aggregation propensity profiles of wild type SGSH (gray), and Arg245His SGSH (red). For clarity only a region containing Arg245His mutation (red dot) is shown. (C) Aggregation propensity profiles of wild type SGSH (gray) and SGSH Ser66Trp (purple). For clarity only a region containing Ser66Trp mutation (purple dot) is shown. The square peaks in each profile represent the significant Hot Spot (HS) areas in the protein sequence. Profiles were created using the AGGRESCAN algorithm [6]. (TIF) [file pone.0121511.s003.tif]

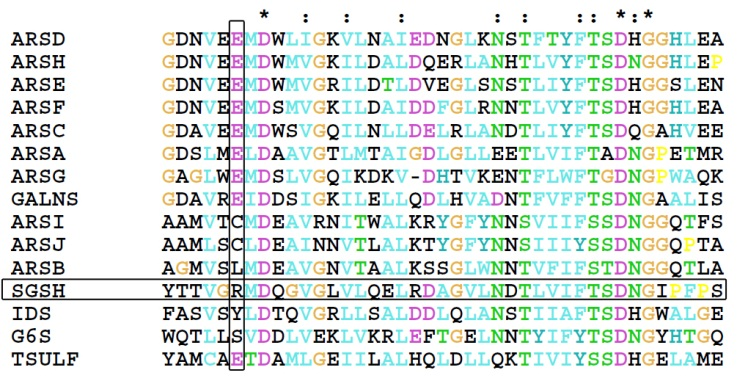

Supplement: S3 Fig — Alignment was performed with ClustalX2 (default software color-coding was used). Only a small portion of the sequence alignment showing the relevant region for the amino acid residue Arg245 is shown for clarity. SGSH sequence is shown in the horizontal rectangle. The position of the Arginine at position 245 is indicated using a vertical rectangle. The annotation of the sulfatases is used as outlined in [14]. The stars denote residues of identity in all of the related protein sequences. Colons are used to indicate those amino acid positions where the residues show high conservation (amino acids with similar physico-chemical properties). (TIF) [file pone.0121511.s004.tif]
