# Supplementary material for: A Multiparametric Computational Algorithm for Comprehensive Assessment of Genetic Mutations in Mucopolysaccharidosis Type IIIA (Sanfilippo Syndrome)
Source: PLoS One. 2015 Mar 25;10(3):e0121511. doi: 10.1371/journal.pone.0121511 (PMC4373678; doi:10.1371/journal.pone.0121511)
Supplement: S1 Table — *Patient ID is according to the cited paper. (DOCX) [file pone.0121511.s005.docx]

| **Mutation** | **Age of Diagnosis**  **year/mo.** | **Mutation Total Score Mutation** | **Reference** | **Patient ID*** |
| --- | --- | --- | --- | --- |
| Ser66Trp | 3/5 | 4 | 19 | Patient 21 |
| Ser66Trp | 4/0 | 4 | 20 | Patient 29 |
| Arg234Gly | 5/0 | 2 | 20 | Patient 18 |
| Arg245His | 2/8 | 5 | 20 | Patient 24 |
| Arg245His | 1/7 | 5 | 20 | Patient 25 |
| Arg245His | 4/10 | 5 | 20 | Patient 30 |
| Arg245His | fetus | 5 | 20 | Patient 11 |
| Arg245His | 3/0 | 5 | 19 | Patient 1 |
| Arg245His | 1/0 | 5 | 19 | Patient 3 |
| Arg245His | 3/1 | 5 | 19 | Patient 7 |
| Pro288Ser | 3/0 | 2 | 21 | Patient 4 |
