# Supplementary material for: A Multiparametric Computational Algorithm for Comprehensive Assessment of Genetic Mutations in Mucopolysaccharidosis Type IIIA (Sanfilippo Syndrome)
Source: PLoS One. 2015 Mar 25;10(3):e0121511. doi: 10.1371/journal.pone.0121511 (PMC4373678; doi:10.1371/journal.pone.0121511)
Supplement: S2 Table — If a mutation was referred to as mild/intermediate it was given an overall assessment of intermediate. If a mutation was referred to as intermediate/severe, it was given an overall assessment of severe. *Patient ID is according to the cited paper. ** Severity is assumed from early death of patient caused by MPS-IIIA disease (12 years old) [27]. (DOCX) [file pone.0121511.s006.docx]

| **Mutation** | **Severity** | **Mutation**  **Total Score** | **Reference** | **Patient ID*** |
| --- | --- | --- | --- | --- |
| Ser66Trp | severe | 4 | 22 | Patient S.D. |
| Ser66Trp | intermediate | 4 | 22 | Patient G.A. |
| Ser66Trp | intermediate | 4 | 22 | Patient M.C. |
| Ser66Trp | severe | 4 | 22 | Patient Si. M. |
| Ser66Trp | severe | 4 | 22 | Patient F. P. |
| Ser66Trp | severe | 4 | 20 | Patient 29 |
| His84Tyr | severe | 6 | 23 | Patient 4 |
| Gln85Arg | severe | 3 | 24 | Patient SFA-11 |
| Gly122Arg | intermediate | 7 | 22 | Patient C.F. |
| Leu146Pro | severe | 4 | 22 | Patient C.T. |
| Arg150gln | severe | 6 | 22 | Patient Pe.N. |
| Arg206Pro | intermediate | 4 | 25 | Patient R.M. |
| Ala234Gly | severe | 2 | 20 | Patient 18 |
| Arg245His | severe | 5 | 26 | Patient 3348 |
| Arg245His | severe | 5 | 23 | Patient 11 |
| Pro288Ser | severe | 2 | 21 | Patient4 |
| Ser298Pro | mild | 3 | 27 | patient 73.1 |
| Ser298Pro | mild | 3 | 27 | patient 20.1 |
| Ser298Pro | mild | 3 | 27 | patient 41.1 |
| Ser298Pro | mild | 3 | 27 | patient 41.2 |
| Ile322Ser | mild | 2 | 23 | Patient 8 |
| Ser347Tyr | severe** | 5 | 27 | Patient 27.1 |
| Arg377Cys | severe | 6 | 22 | Patient C.A. |
| Leu386Arg | severe | 5 | 24 | Patient SFA-6 |
| Arg433Trp | severe | 5 | 28 | Patient SFA-14 |
| Glu447Lys | severe | 4 | 28 | Patient SFA-10 |
| Glu447Lys | severe | 4 | 28 | Patient SFA-16 |
| Val486Phe | severe | 6 | 23 | Patient 17 |
