# Supplementary material for: A Multiparametric Computational Algorithm for Comprehensive Assessment of Genetic Mutations in Mucopolysaccharidosis Type IIIA (Sanfilippo Syndrome)
Source: PLoS One. 2015 Mar 25;10(3):e0121511. doi: 10.1371/journal.pone.0121511 (PMC4373678; doi:10.1371/journal.pone.0121511)
Supplement: S3 Table — If a mutation was referred as mild/intermediate it was assigned a value of intermediate. If a mutation was referred as intermediate/severe, it was assigned a value of severe. *Patient ID is according to the cited paper.**Severity is assumed from the current age at the clinical examination and the explanation of the reports for patients bearing S298P mutations (alive 36 years patient) [27]. ^Data not included in analysis. Val131Met is the only mutation in our set that has a total score of 0, which does not allow calculations for compound heterozygous individuals. (DOCX) [file pone.0121511.s007.docx]

| **Mutation 1** | **Mutation 2** | **Severity** |  | **Total Score Mutation 1** | **Total Score Mutation 2** | **Compound score** | **Reference** | **Patient ID*** |
| --- | --- | --- | --- | --- | --- | --- | --- | --- |
| Ser66Trp | Arg245His | severe |  | 4 | 5 | 20 | 26 | Patient 2804 |
| Ser66Trp | Arg245His | severe |  | 4 | 5 | 20 | 20 | Patient 3 |
| Ser66Trp | Ser298Pro | intermediate |  | 4 | 3 | 12 | 27 | Patient 25.1 |
| Thr79Pro | Arg245His | severe |  | 5 | 5 | 25 | 20 | Patient 36 |
| Ser106Arg | Arg245His | intermediate |  | 5 | 5 | 25 | 29 | n/a |
| Pro128Leu | Glu369Lys | intermediate |  | 6 | 5 | 30 | 22 | Patient S.M. |
| Val131Met | Ser66Trp | severe |  | 0 | 4 | 0^ | 20 | Patient 37 |
| Arg150Gln | Ser66Trp | severe |  | 6 | 4 | 24 | 24 | Patient SFA-9 |
| Leu163Pro | Arg245His | severe |  | 6 | 5 | 30 | 29 | n/a |
| Pro180Leu | Arg245His | mild |  | 4 | 5 | 20 | 27 | Patient 31.1 |
| Gly191Arg | Arg245His | severe |  | 4 | 5 | 20 | 29 | n/a |
| Arg245His | Ser66Trp | severe |  | 5 | 4 | 20 | 20 | Patient 42 |
| Arg245His | Ser298Pro | mild |  | 5 | 3 | 15 | 30 | Patient SF 1 |
| Arg245His | Gln380Arg | severe |  | 5 | 3 | 15 | 20 | Patient 38 |
| Arg245His | Ser66Trp | severe |  | 5 | 4 | 20 | 23 | Patient 9 |
| Arg245His | Ser298Pro | Intermediate |  | 5 | 3 | 15 | 27 | Patient 17.1 |
| Arg245His | Ser298Pro | Intermediate |  | 5 | 3 | 15 | 27 | Patient 23.1 |
| Arg245His | Ser298Pro | Intermediate |  | 5 | 3 | 15 | 27 | Patient 23.2 |
| Arg245His | Ser298Pro | Intermediate |  | 5 | 3 | 15 | 27 | Patient 23.3 |
| Arg245His | Ser298Pro | Intermediate |  | 5 | 3 | 15 | 27 | Patient 33.1 |
| Arg245His | Ser298Pro | Intermediate |  | 5 | 3 | 15 | 27 | Patient 33.2 |
| Arg245His | Ser298Pro | Intermediate |  | 5 | 3 | 15 | 27 | Patient 34.2 |
| Arg245His | Ser298Pro | Intermediate |  | 5 | 3 | 15 | 27 | Patient 38.1 |
| Arg245His | Ser298Pro | Intermediate |  | 5 | 3 | 15 | 27 | Patient 38.2 |
| Arg245His | Ser298Pro | Intermediate |  | 5 | 3 | 15 | 27 | Patient 40.1 |
| Arg245His | Ser298Pro | intermediate |  | 5 | 3 | 15 | 27 | Patient 42.1 |
| Arg245His | Ser298Pro | Intermediate |  | 5 | 3 | 15 | 27 | Patient 48.1 |
| Arg245His | Ser298Pro | intermediate |  | 5 | 3 | 15 | 27 | Patient 48.2 |
| Arg245His | Ser298Pro | intermediate |  | 5 | 3 | 15 | 27 | Patient 49.1 |
| Arg245His | Ser298Pro | intermediate |  | 5 | 3 | 15 | 27 | Patient 52.1 |
| Arg245His | Ser298Pro | intermediate |  | 5 | 3 | 15 | 27 | Patient 57.1 |
| Arg245His | Ser298Pro | intermediate |  | 5 | 3 | 15 | 27 | Patient 57.2 |
| Arg245His | Ser298Pro | intermediate |  | 5 | 3 | 15 | 27 | Patient 57.3 |
| Arg245His | Ser298Pro | intermediate |  | 5 | 3 | 15 | 27 | Patient 70.1 |
| Arg245His | Ser298Pro | intermediate |  | 5 | 3 | 15 | 27 | Patient 70.2 |
| Arg245His | Ser298Pro | intermediate |  | 5 | 3 | 15 | 27 | Patient 74.1 |
| Arg245His | Ser298Pro | intermediate |  | 5 | 3 | 15 | 27 | Patient 86.1 |
| Arg245His | Ser298Pro | intermediate | | 5 | 3 | 15 | 27 | Patient 89.1 |
| Ser298Pro | Thr421Arg | mild |  | 3 | 3 | 9 | 27 | Patient 75.1 |
| Ser298Pro | Thr421Arg | mild |  | 3 | 3 | 9 | 27 | Patient 75.2 |
| Ser298Pro | Leu411Arg | mild** |  | 3 | 3 | 9 | 27 | Patient 55.1 |
| Gln380Arg | Arg245His | severe |  | 3 | 5 | 15 | 27 | Patient 53.1 |
| Arg433Trp | Arg245His | severe |  | 5 | 5 | 25 | 23 | Patient 3 |
| Glu447Lys | Arg245His | severe |  | 4 | 5 | 20 | 26 | Patient 3 |
