# Supplementary material for: A Multiparametric Computational Algorithm for Comprehensive Assessment of Genetic Mutations in Mucopolysaccharidosis Type IIIA (Sanfilippo Syndrome)
Source: PLoS One. 2015 Mar 25;10(3):e0121511. doi: 10.1371/journal.pone.0121511 (PMC4373678; doi:10.1371/journal.pone.0121511)
Supplement: S4 Table — Analysis was performed with GraphPad Prizm software. (DOCX) [file pone.0121511.s008.docx]

|  | **Correlation coefficient (r)** | **p**  **(two tailed)** | **n** |
| --- | --- | --- | --- |
| Compound score as a sum | 0.57 | <0.0001 | 44 |
| **Compound score as a product** | **0.67** | **<0.0001** | **43** |
